# Antibiotic Resistance Profile of Wound Infection Isolates: High Resistance to Cephalosporins and Penicillin Among Ghanaian Patients in a Cross‐Sectional Study

**DOI:** 10.1002/hsr2.72176

**Published:** 2026-05-28

**Authors:** Nelson Kwashie, Samuel Antwi‐Baffour, Lawrence Annison, Lydia Ofosua Appah, Uwakmfon Paul, Solomon Akpobi, Ebenezer Senu

**Affiliations:** ^1^ Department of Medical Laboratory Science, School of Allied Health Sciences Narh‐Bita College Tema Greater Accra region Ghana; ^2^ Department of Medical Diagnostics, Faculty of Allied Health Sciences Kwame Nkrumah University of Science and Technology Kumasi Ashanti region Ghana; ^3^ Laboratory Department, School of Public Health, KNUST‐IVI Collaboration Center Kwame Nkrumah University of Science and Technology Kumasi Ashanti region Ghana; ^4^ Department of Clinical Research and Data Science Elite Research and Data Science Institute Kumasi Ashanti Region Ghana; ^5^ Department of Molecular Medicine, School of Medicine and Dentistry Kwame Nkrumah University of Science and Technology Kumasi Ashanti region Ghana; ^6^ Department of Biological Sciences, School of Natural Sciences and Mathematics The University of Texas at Dallas Richardson Texas USA

**Keywords:** aminoglycosides, antimicrobial resistance (AMR), cephalosporins, *Escherichia coli*, Klebsiella spp., linezolid, penicillin, *Pseudomonas aeruginosa*, *Staphylococcus aureus*

## Abstract

**Background and Aim:**

Antibiotics remain among the most frequently prescribed medications in clinical practice. However, antimicrobial resistance (AMR) is an inevitable and irreversible consequence of bacterial adaptation to antimicrobial exposure. Despite its clinical importance, data on antibiotic resistance patterns of bacteria from wound infections in Ghana are limited. This study evaluated the prevalence and magnitude of antibiotic resistance among bacterial isolates from infected wounds in a Ghanaian population.

**Methods:**

This experimental cross‐sectional study enrolled 300 patients with suspected wound infections receiving care at MDS‐Lancet Laboratories. Wound swabs were collected, cultured on standard media, and incubated at 37°C for 18–24 h, with a second overnight reading. Bacterial identification was based on colony morphology, Gram staining, biochemical testing, and API 20 E/NE. Antimicrobial susceptibility was determined using the disc diffusion method. Data analysis was conducted using SPSS v26.0 and GraphPad v9.5.

**Results:**

The predominant isolates were *Escherichia coli* (23.2%), *Pseudomonas aeruginosa* (19.8%), *Klebsiella* spp. (19.2%), and *Staphylococcus aureus* (15.2%). Gram‐negative isolates were most susceptible to aminoglycosides (Amikacin, Gentamicin), carbapenems (Ertapenem, Imipenem, Meropenem), and Tigecycline, whilst showing high resistance to 2nd, 3rd and 4th generation cephalosporins (Cefuroxime, Ceftriaxone, and Cefepime, respectively) and Amoxicillin. Gram‐positive isolates, including *S. aureus* and coagulase‐negative staphylococci, were most susceptible to Gentamicin, Clindamycin, and Erythromycin, but resistant to 1st‐ and 2nd‐generation cephalosporins (Cefazolin and Cefoxitin respectively), penicillins (Augmentin and Cloxacillin), and Tetracycline. Extended‐spectrum β‐lactamase (ESBL) producers accounted for 30.3% of isolates, predominantly *E. coli* (50%) and *Klebsiella* spp. (43.9%). Methicillin‐resistant *S. aureus* (MRSA) and methicillin‐resistant coagulase‐negative staphylococci (MRCNS) were observed in 34.7% and 66.7% of isolates, respectively.

**Conclusion:**

Aminoglycosides and Linezolid are most effective against Gram‐positive and Gram‐negative bacteria causing wound infections. However, both Gram‐positive and Gram‐negative bacteria are resistant to 2nd generation cephalosporins and penicillin. High resistance to cephalosporins and penicillin calls for the necessity of culture‐guided therapy for effective wound infection management.

## 1 Introduction

Wound infection is one of the major health problems in the world due to its ability to pose serious and disastrous complications that result in many deaths. The skin is in contact with the external environment, and it serves as a defense barrier against the colonization of pathogens or a protective layer for the underlying organs or structures [1]. The disruption of the normal anatomical structure, either by surgical operations or by chemical, physical, or thermal events (burns), with an alteration of skin functions, results in a wound [2, 3]. Wound infections represent one third of nosocomial infections among surgical patients and are responsible of 70%–80% of mortality in patients, especially in developing countries, regardless of the type of wound [4, 5, 6]. In the USA alone, 6.5 million wounds with evidence of bacterial infection are diagnosed every year [7]. The WHO projects the terrifying prospect of 10 million AMR‐related deaths per year globally by 2050.

A number of factors contribute to wound infections; however, microorganisms are the major cause, with bacteria being the most prevalent [8]. Wound infection can be caused by a single bacterium or multiple microorganisms. Hard‐to‐heal wounds such as venous ulcers, pressure ulcers, and diabetic foot ulcers are usually infected by multiple microorganisms [9, 10, 11], which may exist predominantly in the form of a biofilm resistant to antimicrobial treatments. Early recognition of wound infection and appropriate management is thus important. Antibiotic therapy and surgical management are the cornerstone measures whereby antibiotics offer adjuvant treatment. A number of studies indicate an increase in antibiotic resistance globally [4]. The mechanism by which resistance develops is complex and can result in multidrug‐resistant bacterial strains due to the simultaneous development of resistance to several antibiotics. Moreover, the lack of new antibodies to replace old ones to which resistance has been developed further increases the challenges posed by AMR. The absence of a proper monitoring and regulatory framework in the use of antimicrobial agents, inadequate infection control practices, and limited resources in the diagnosis of infectious diseases have contributed to the worldwide development of AMR. In low and middle‐income countries, especially in sub‐Saharan Africa, inadequate policies have resulted in the irrational use and abuse of antibiotics in almost all settings where they are needed [12]. After half a century of antibiotic use, the emergence and spread of bacterial resistance is a critical public health issue and is growing in importance worldwide, especially in hospitals, and has now reached higher proportions in the world. The extensive use of broad‐spectrum antibiotics in human medicine to treat infections without diagnosis of the specific pathogen involved is a contributing factor [13]. In 2015, global leaders at the World Health Assembly authorized a global action plan to tackle AMR in the world. This culminated in the first‐ever “African conference on antibiotic use and resistance” held in Ghana in March 2015 to publicize research information on AMR within the African sub‐regions.

The World Health Organization (WHO) has considered antimicrobial resistance as one of the top 10 threats to global health. The growing menace of antibiotic resistance is more worrying against the backdrop of the limited discovery of new antibiotics. Antibiotic treatment and wound care represent two critical factors for the management of the infection. More than half of the admitted patients receive one or more antibiotics in Ghana. However, there is a limited study on the antibiotic resistance pattern of bacteria isolated from infected wounds. The detection of microbial species pathogens' distribution and antimicrobial susceptibility patterns is often underestimated. In this study, we analyzed antibiotic resistance patterns among bacteria isolated from infected wounds and observed widespread resistance to first‐line antibiotics commonly used in clinical practice among Ghanaians in the Greater Accra region. Findings from this study will not only contribute to existing knowledge but will also inspire further research into this area, which shall help to develop a better protocol for treatment and also help policy makers to have enough information on antimicrobial resistance (AMR) in Ghana.

## 2 Materials and Methods

### 2.1 Study Design

This study adopted an experimental cross‐sectional study design. A cross‐sectional study design is an observational study in which a researcher concurrently measures the exposures and outcome in a study population based on the inclusion and exclusion criteria for the study [14]. This study involved using a sterile swab with transport medium to take wound samples from study participants and sent them to the lab for microbiological analyses.

### 2.2 Study Site

This study was carried out in the microbiology unit of the clinical laboratory unit of MDS‐Lancet Laboratories located in East Legon, Accra‐Ghana. MDS Lancet Laboratories Ghana Ltd. is a member of the Lancet Laboratories group, which was founded over 60 years ago and operates in about 11 African countries, including Ghana. It has a well‐equipped microbiology unit, making it suitable for the successful implementation of the study.

### 2.3 Study Participants

Participants were enrolled from walking‐in patients or referred patients with wound infection cases between the study period, receiving health care at Lancet Laboratories. The diagnosis of infection was based on the criteria stated by the Center for Disease Control and Prevention [15].

### 2.4 Inclusion and Exclusion Criteria

Patients with suspected wound infection, who present with purulent exudate, serous exudate, foul smell, signs of inflammation, edema, discolored granulation tissue, and friable granulation tissue, visiting the facility within the study period, and who gave consent were included in the study. However, patients who did not give consent or had other pus cases that were not from wound sources were excluded from the study.

### 2.5 Sample Size Calculation

The sample size is obtained using the formula:

*n* = $\frac{Z^{2}\mathrm{PQ}}{d^{2}}$; Where: *n* is the minimum sample size, *P* is prevalence of infected wounds in the population estimated to be 22%, *Q* = 1 −* P*, *Z *= *z* value at 95% confidence (1.96), and *d* is the margin of error (0.05)

$$n\;\left(\text{Minimum number of participants}\right)=\frac{1.96^{2}(0.22)\left(1-0.22\right)}{0.05^{2}}=264$$

Hence, a minimum of 264 participants was required for the study. To increase statistical power, three hundred (300) participants were recruited for the study, of which three (3) had no growth.

### 2.6 Ethical Consideration

Ethical approval was obtained from the ethics and protocol review committee of Narh Bita College before the study was conducted. A detailed explanation of the study protocol and assurance of anonymity were provided to the subjects. Participants were then given written informed consent, which was read and explained to them in a language they understood, to consent before participating in this study. The study was conducted following the guidelines of the Helsinki Declaration.

### 2.7 Sociodemographic and Clinical Data Collection

Participants' sociodemographic and clinical data were captured using a well‐structured questionnaire. This included sociodemographic data such as age, gender, place of residence, and clinical data such as presence of comorbidity, history of antibiotic use, type of antibiotic used before, history of chronic diseases or infection.

### 2.8 Sample Collection

Wound swabs were taken from the infected site of the wounds using sterile cotton swabs, rotating the swab in a “Z” movement, avoid touching the surrounding skin and finally the swab was reinserted into the appropriate tube containing the Amies transport medium and transported on ice to the microbiology department within 30 min to 1 h to increase the chance of isolating the causative agent.

### 2.9 Laboratory Analysis

All samples received for the study were registered, and the swaps were inoculated onto Blood agar (BA), MacConkey agar (MA), Chocolate agar (CA), and Sabouraud agar (SA) plates. Using an inoculating loop, each sample was streaked onto the upper one‐fourth portion of an agar plate (labeled with the patient's ID) with parallel overlapping strokes. The loop was flame and allowed to cool. The plate was turned at a right angle and streaked twice, overlapping the previous ones. The BA, MA, and SA were incubated at 37°C in an aerobic condition, whilst CA was incubated in a jar containing co₂ gas pack for 18–24 h and examined for growth and further re‐incubation for another 18–24 h for samples that showed no growth or growth was not profound. The swabs were also inoculated into thioglycolate broth, which was resubbed the next day. Wet preparations were also done for the wound swabs to quantify pus cells as the number of cells/High power field (Hpf). Results interpreted as none (0/Hpf), few (1–4/Hpf), moderate (5–9/Hpf), and many (> 10/Hpf).

Identification of the isolates was done by Gram staining and based on colonial morphology characteristics on the plates, such as hemolysis on blood agar, swarming (positive for *Proteus* spp.), and changes in physical appearance on differential media. Standard biochemical tests were performed as described by [16] to identify the species. API 20E/NE was also done to help in the identification process.

### 2.10 Antibiotics Susceptibility Testing (AST)

Antimicrobial susceptibility Testing (AST) is an important test in the microbiology laboratory which informs the susceptibility of microorganisms to specific antibiotics. Antimicrobial resistance is the ability of a microbe to survive and multiply in the presence of an antimicrobial agent that would normally inhibit or have a suicidal activity against the microbe.

### 2.11 Disk Diffusion Method of Antimicrobial Susceptibility Testing

Antibiotic susceptibility testing was done using Kirby–Bauer's disc diffusion method according to the Clinical and Laboratory Standards Institute (CLSI) guidelines [17]. It is a simple and well‐standardized test where a plate of Muller–Hinton agar was streaked with an inoculum of a 0.5 McFarland equivalent suspension of the organism using a sterile cotton swab at three different angles to give a confluent growth as described in the CLSI recommendations. Up to six already commercially prepared, fixed concentration paper antibiotic disks were placed on the inoculated agar surface and incubated for 18–24 h at 37°C before results were taken. The diameter of zones of inhibition of growth around each of the antibiotic disks was measured to the nearest millimeter. The diameter was related to the susceptibility of the isolate and the rate of diffusion through the agar medium. The diameters of each zone were interpreted using the criteria published by CLSI. The results obtained were recorded as susceptible, intermediate, or resistant.

### 2.12 Multidrug‐Resistant (MDR) Bacteria Strains

Isolates that showed resistance to ≥ 1 antimicrobial agent in ≥ 3 different antimicrobial classes were described as multidrug‐resistant (MDR) strains.

### 2.13 Quantification of Bacterial Isolates

Quantification of bacteria isolates was done using the “3‐phase streaking techniques”. Blood and MacConkey plates were divided into four sections. Sterile loops dipped into 0.5 mL prepared inoculums were used to streak the quadrants, starting from the first to the fourth, without dipping them back into the inoculums. The plates were incubated aerobically at 37°C overnight for approximately 18 h. Bacteria were described as “few”, “moderate”, and “many” with respect to growth in the first, second, and third or fourth quadrants, respectively.

Quality control test for both media and antibiotic disks used was carried out using *Escherichia coli* control strain (ATCC 25022) and *Staphylococcus aureus* (ATCC).

### 2.14 Data Management and Statistical Analysis

Collected data was entered, cleaned, and coded using Microsoft Excel 2019. All statistical analyses were done using the Statistical Package for Social Sciences (SPSS) Version 26.0 (Chicago, IL, USA) and GraphPad Prism version 8.0 (GraphPad software, San Diego, California, USA, www.graphpad.com). Categorical variables were presented as frequencies and percentages. A bar chart was used to illustrate the distribution of age and gender, antibiogram, bacteria isolates, prevalence of ESBL, and MRSA. A pie chart was also used to illustrate the distribution of ESBL and MRSA bacteria. Inferential statistics were done by *χ* ^2^. *p* value of < 0.05 was considered statistically significant.

## 3 Results

### 3.1 Age and Gender Distribution of Wound Patients With Positive Bacterial Wound Infection

**Figure 1:**
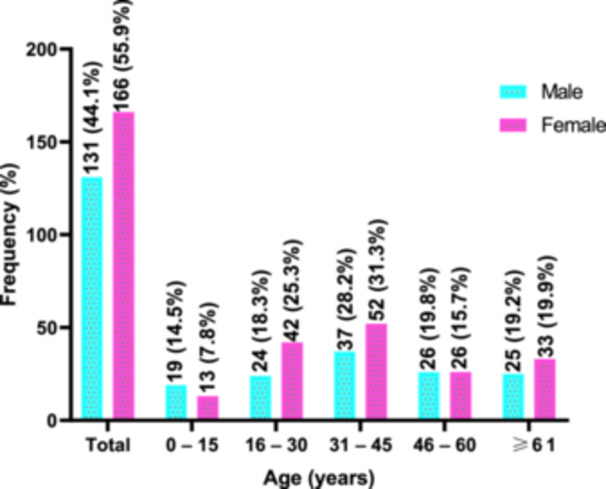
Age and gender distribution of wound patients with positive bacterial wound infection.

Of the study participants, more than half were females (55.9%), whilst 44.1% were males. The majority of female participants were within the ages of 31–45 years (31.3%), followed by 16–30 years (25.3%), and more than 60 years (19.9%). Of the male participants, the majority were within 31–45 years (28.2%), followed by more than or equal to 61 years (19.2%), and 16–30 years (18.3%) (Figure 1).

### 3.2 Prevalence of Organisms Causing Wound Infections Among Study Participants

**Figure 2:**
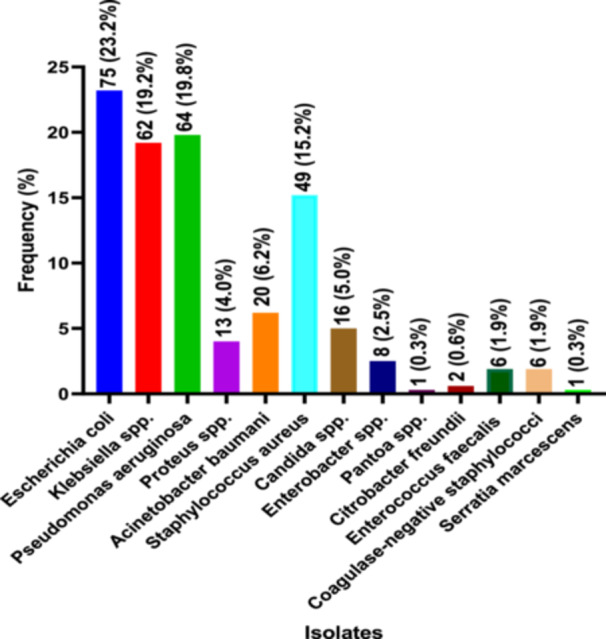
Prevalence of organisms causing wound infections among study participants.

Of organisms causing wound infections isolated, most were *E. coli* (23.2%), followed by *P. aeruginosa* (19.8%), *Klebsiella* spp. (19.2%), and *Staphylococcus aureus* (15.2%). A significant number were also *Acinetobacter baumani* (6.2%), *Candida* spp. (5.0%), *Proteus* spp. (4.0%), and *Enterobacter* spp. (2.5%). Few were coagulase‐negative staphylococci (CNS) (1.9%), *Enterococcus faecalis* (1.9%), *Citrobacter freundii* (0.6%), *Enterobacter* spp. (0.3%), and *Serratia marcescens* (0.3%) (Figure 2).

### 3.3 Gender Distribution of Isolated Organisms Causing Wound Infections Among Study Participants

Of the isolates, *E. coli* (24.0%), *P. aeruginosa* (20.6%), *Candida* spp. (6.3%), *Enterobacter* spp. (2.9%), and *E. faecalis* (2.9%) were predominantly found in the female participants. Whereas *A. baumani* (8.1%), *S. aureus* (16.2%), and coagulase‐negative staphylococci (CNS) (3.4%) were predominantly found in male participants (3.4%). A significant difference was therefore found in the distribution of isolated organisms causing wound infections stratified by gender among study participants (*p* < 0.0001) (Table 1).

**Table 1:** Gender distribution of isolated organisms causing wound infections among study participants.

	Total (*n* = 323)	Male (*n* = 148)	Female (*n* = 175)	*p* value
Isolates				< 0.0001
*Escherichia coli*	75 (23.2)	33 (22.3)	42 (24.0)	
*Klebsiella* spp.	62 (19.2)	31 (20.9)	31 (20.9)	
*Pseudomonas aeruginosa*	64 (19.8)	28 (18.9)	36 (20.6)	
*Proteus* spp.	13 (4.0)	4 (2.7)	9 (5.1)	
*Acinetobacter baumani*	20 (6.2)	12 (8.1)	8 (4.6)	
*Staphylococcus aureus*	49 (15.2)	24 (16.2)	25 (14.3)	
*Candida* spp.	16 (5.0)	5 (3.4)	11 (6.3)	
*Enterobacter* spp.	8 (2.5)	3 (2.0)	5 (2.9)	
*Pantoa* spp.	1 (0.3)	0 (0.0)	1 (0.6)	
*Citrobacter freundii*	2 (0.6)	1 (0.7)	1 (0.6)	
*Enterococcus faecalis*	6 (1.9)	1 (0.7)	5 (2.9)	
Coagulase‐negative staphylococci (CNS)	6 (1.9)	5 (3.4)	1 (0.6)	
*Serratia marcescens*	1 (0.3)	1 (0.7)	0 (0.0)	

*Note:* Data presented as frequency and percentage. *p*‐value computed by Chi‐square test.

### 3.4 Resistance Pattern of Enterobacteriaceae Causing Wound Infections in This Study

Thirteen (13) antibiotics belonging to six different classes including aminoglycoside (AMK: Amikacin, GENT: Gentamicin), 2nd generation cephalosporin (CFM: Cefuroxime) 3rd generation cephalosporin (CRO: Ceftriaxone) 4th generation cephalosporin (CFPM: Cefepime), fluoroquinolone (CIP: Ciprofloxacin), aminopenicillin (AUG: Amoxicillin, PTZ: Piperacillin Tazobactam), tetracycline (TG: Tigecycline), sulfonamides‐trimethoprim (COTRI: Trimethoprim), carbapenem (ERT: Ertapenem, IMI: Imipenem, MEM: Meropenem) were tested on *Klebsiella* spp., *C. fruendii*, *Pantoa* spp., *Enterobacter* spp., *E. coli* and *S. marscensence*; whilst eleven (11) antibiotics (AMK, CXM, CFPM, CRO, CIP, AUG, COTRI, ERT, GENT, MEM, PTZ) were tested on *Proteus* spp.

Of the 62 *Klebsiella spp*. isolates, the highest resistance was against AUG (79.0%), followed by CXM (74.2%), COTRI (73.8%), PTZ (72.6%), CFPM (69.4%), CRO (69.4%), CIP (64.5%) and GENT (48.4%). Of both *C. fruendii*, and *Pantoa spp*. isolates had the highest resistance against COTRI (100.0%; 100.0%), CXM (50.0%; 100.0%), CFPEM (50.0%; 100.0%), CRO (50.0%; 100.0%), CIP (50.0%; 100.0%), AUG (50.0%; 100.0%), and PTZ (50.0%; 100.0%). Moreover, of 8 *Enterobacter spp*. isolates, the highest resistance was against CXM (75.0%), CRO (75.0%), AUG (75.0%), COTRI (50.0%), and CIP (37.5%). Of 75 *E. coli* isolates, the highest resistance was found against AUG (81.3%), COTRI (78.7%), CXM (73.3%), PTZ (69.3%), CIP (68.0%), CRO (66.7%), CFPM (65.3%), and GENT (37.3%). CXM (100.0), CRO (100.0), and AUG (100.0) were also resistant against *S. marscensence* isolate. For 13 *Proteus spp*. isolated, most were resistant against COTRI (69.2%), CIP (38.5%), AUG (38.5%), GENT (30.8%), and some were resistant against CXM (23.1%), CFPM (23.1%), CRO (23.1%), and PTZ (23.1%) (Table 2).

**Table 2:** Antibiogram of GNBs (Enterobacteriaceae) isolated in this study.

Antibiotics	*Klebsiella* spp. (*n* = *62)*		*C. fruendii (n* = *2)*		*Pantoa* spp. (*n* = *1)*		*Enterobacter* spp. (*n* = *8)*		*E. coli (n* = *75)*		*S. marscensence (n* = *1)*		*Proteus* spp. (*n* = *13)*	
	*R*	*S*	*R*	*S*	*R*	*S*	*R*	*S*	*R*	*S*	*R*	*S*	*R*	*S*
AMK	7 (11.3)	55 (88.7)	0 (0.0)	2 (100.0)	0 (0.0)	1 (100.0)	0 (0.0)	8 (100.0)	2 (2.7)	73 (97.3)	0 (0.0)	1 (100.0)	0 (0.0)	13 (100.0)
CXM	46 (74.2)	16 (25.8)	1 (50.0)	1 (50.0)	1 (100.0)	0 (0.0)	6 (75.0)	2 (25.0)	55 (73.3)	20 (26.7)	1 (100.0)	0 (0.0)	3 (23.1)	10 (76.9)
CFPM	43 (69.4)	19 (30.6)	1 (50.0)	1 (50.0)	1 (100.0)	0 (0.0)	1 (12.5)	7 (87.5)	49 (65.3)	26 (34.7)	0 (0.0)	1 (100.0)	3 (23.1)	10 (76.9)
CRO	43 (69.4)	19 (30.6)	1 (50.0)	1 (50.0)	1 (100.0)	0 (0.0)	6 (75.0)	2 (25.0)	50 (66.7)	25 (33.3)	1 (100.0)	0 (0.0)	3 (23.1)	10 (76.9)
CIP	40 (64.5)	22 (35.5)	1 (50.0)	1 (50.0)	1 (100.0)	0 (0.0)	3 (37.5)	5 (62.5)	51 (68.0)	24 (32.0)	0 (0.0)	1 (100.0)	5 (38.5)	8 (61.5)
AUG	49 (79.0)	13 (21.0)	1 (50.0)	1 (50.0)	1 (100.0)	0 (0.0)	6 (75.0)	2 (25.0)	61 (81.3)	14 (18.7)	1 (100.0)	0 (0.0)	5 (38.5)	8 (61.5)
COTRI	45 (73.8)	16 (26.8)	2 (100.0)	0 (0.0)	1 (100.0)	0 (0.0)	4 (50.0)	4 (50.0)	59 (78.7)	16 (21.3)	0 (0.0)	1 (100.0)	9 (69.2)	4 (30.8)
ERT	6 (9.7)	56 (90.3)	0 (0.0)	2 (100.0)	0 (0.0)	1 (100.0)	0 (0.0)	8 (100.0)	2 (2.7)	73 (97.3)	0 (0.0)	1 (100.0)	2 (15.4)	11 (84.6)
GENT	30 (48.4)	32 (51.6)	0 (0.0)	2 (100.0)	0 (0.0)	1 (100.0)	1 (12.5)	7 (87.5)	28 (37.3)	47 (62.7)	0 (0.0)	1 (100.0)	4 (30.8)	9 (69.2)
IMI	5 (8.1)	57 (91.9)	0 (0.0)	2 (100.0)	0 (0.0)	1 (100.0)	0 (0.0)	8 (100.0)	1 (1.3)	74 (98.7)	0 (0.0)	1 (100.0)	—	—
MEM	7 (11.3)	55 (88.7)	0 (0.0)	2 (100.0)	0 (0.0)	1 (100.0)	0 (0.0)	8 (100.0)	2 (2.7)	73 (97.3)	0 (0.0)	1 (100.0)	2 (15.4)	11 (84.6)
PTZ	45 (72.6)	17 (27.4)	1 (50.0)	1 (50.0)	1 (100.0)	0 (0.0)	1 (12.5)	7 (87.5)	52 (69.3)	23 (30.7)	0 (0.0)	1 (100.0)	3 (23.1)	10 (76.9)
TG	5 (8.1)	57 (91.9)	0 (0.0)	2 (100.0)	0 (0.0)	1 (100.0)	1 (12.5)	7 (87.5)	8 (10.7)	67 (89.3)	0 (0.0)	1 (100.0)	—	—

*Note:* Data presented as frequency and percentage.

Abbreviations: AMK, amikacin; AUG, amoxicillin; CFPM, cefepime; CIP, ciprofloxacin; COTRI, trimethoprim; CRO, ceftriaxone; CXM, cefuroxime; ERT, ertapenem; GENT, gentamicin; IMI, imipenem; MEM, meropenem; PTZ, piperacillin tazobactam; TG, tigecycline.

### 3.5 Resistance Pattern in *Acinetobacter* Spp. and *Pseudomonas aeruginosa* in This Study

Eleven (11) antibiotics belonging to different classes were tested against *Pseudomonas* and *Acinetobacter* spp. Of 20 *Acinetobacter* spp. isolates, the majority were resistant against CFPM (50.0%), CERTAZ (50.0%), CIP (50.0%), PTZ (50.0%), GENT (45.0%), and TOBRA (45.0%). Some were resistant against IMI (25.0%), MEM (25.0%), and AMK (15.0%).

Of the 64 *P. aeruginosa* isolates, some were resistant against CERTAZ (23.4%), CIP (23.4%), PTZ (21.9%), CFPM (20.3%), TOBRA (20.3%), GENT (18.8%), and a few were resistant against IMI (12.5%), MEM (12.5%), and AMK (7.8%) (Table 3).

**Table 3:** Antibiogram pattern of *A. baumani* and *P. aeruginosa* isolated in this study.

	*A. baumani (n* =* 20)*		*P. aeruginosa (n* *= 64)*	
Antibiotics	*R*	*S*	*R*	*S*
AMK	3 (15.0)	17 (85.0)	5 (7.8)	59 (92.2)
CFPM	10 (50.0)	10 (50.0)	13 (20.3)	51 (79.7)
CERTAZ	10 (50.0)	10 (50.0)	15 (23.4)	49 (76.6)
CIP	10 (50.0)	10 (50.0)	15 (23.4)	49 (76.6)
GENT	9 (45.0)	11 (55.0)	12 (18.8)	52 (81.2)
IMI	5 (25.0)	15 (75.0)	8 (12.5)	56 (87.5)
MEM	5 (25.0)	15 (75.0)	8 (12.5)	56 (87.5)
PTZ	10 (50.0)	10 (50.0)	14 (21.9)	50 (78.1)
TOBRA	9 (45.0)	11 (55.0)	13 (20.3)	51 (79.7)

*Note:* Data presented as frequency and percentage.

Abbreviations: AMK, amikacin; CERTAZ, ceftazidime; CFPM, cefepime; CIP, ciprofloxacin; GENT, gentamicin; IMI, imipenem; MEM, meropenem; PTZ, piperacillin tazobactam; TOBRA, tobramycin.

### 3.6 Resistance Pattern in *Enterococcus faecalis* Isolated Among Study Participants

**Figure 3:**
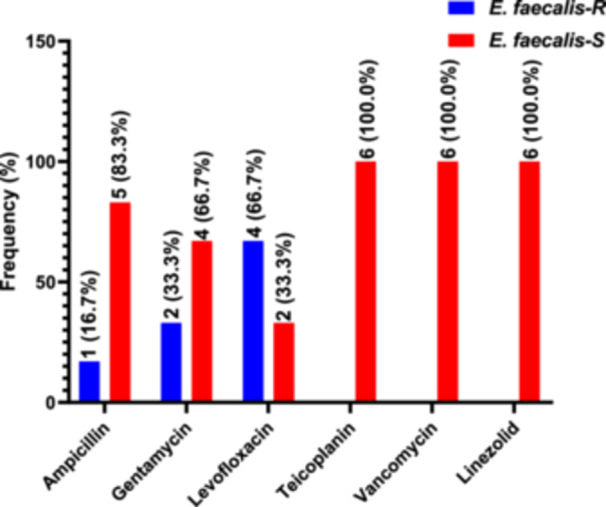
Antibiogram of *Enterococcus faecalis* isolated among study participants.

Of the six (6) *Enterococcus faecalis* isolates tested against six different antibiotics, the highest resistance was against Levofloxacin (66.7%), followed by Gentamicin (33.7%), and some were resistant against Ampicillin (16.7%). None of the participants were resistant to Teicoplanin, Vancomycin, and Linezolid (Figure 3).

### 3.7 Prevalence and Distribution Pattern of ESBL‐Producing Organisms Among Study Participants

**Figure 4:**
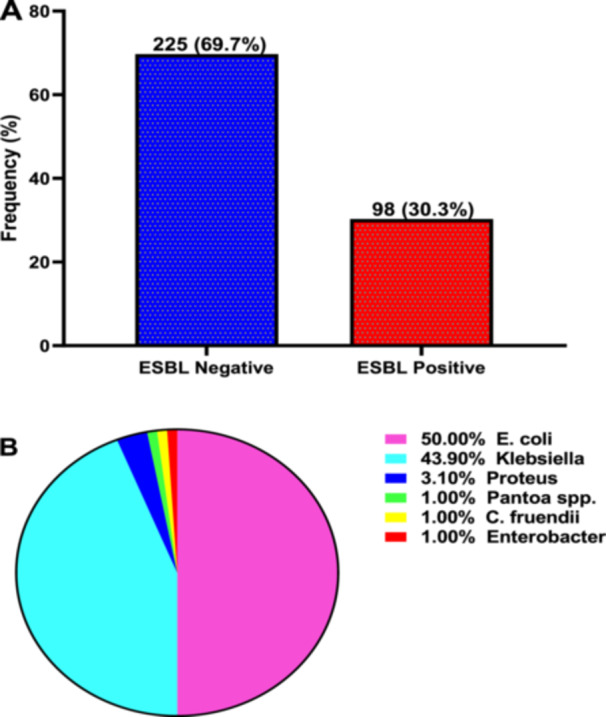
Prevalence (A) and distribution pattern (B) of ESBL‐producing organisms among study participants.

Of the study participants, the prevalence of extended spectrum beta‐lactamase (ESBL) producing organisms isolated was 30.3%, whilst the remaining 225 (69.7%) were ESBL‐negative organisms (Figure 4A).

Of the ESBL‐producing organisms, half were *E. coli* (50.0%), and 43.9% were *Klebsiella* spp. The remaining few ESBL‐positive organisms were *Proteus* spp. (3.1%), *Pantoa* spp. (1.0%), *C. fruendii* (1.0%) and *Enterobacter* spp. (1.0%) (Figure 4B).

### 3.8 Prevalence and Distribution Pattern of Methicillin‐Resistant *Staphylococcus aureus* (MRSA) and Methicillin‐Resistant Coagulase‐Negative *Staphylococcus aureus* (MRCNS)

**Figure 5:**
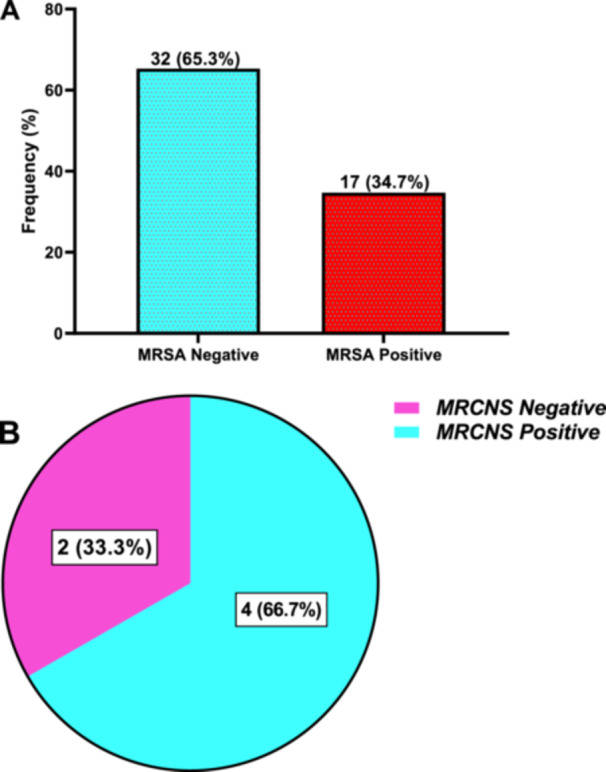
Prevalence and distribution pattern of methicillin‐resistant *Staphylococcus aureus* (MRSA) (A) and coagulase‐negative methicillin‐resistant *S. aureus* (MRCNSA) (B) organisms among study participants.

The prevalence of methicillin‐resistant *S. aureus* (MRSA) positive organisms was 34.7%, whilst the remaining 32 (65.3%) isolates were MRSA negative organisms (Figure 5A).

Moreover, four coagulase‐negative *S. aureus* were methicillin‐resistant, representing 66.7%, whilst the remaining 2 (33.3%) were non‐methicillin‐resistant coagulase‐negative *S. aureus* (Figure 5B).

### 3.9 Resistance Pattern in *S. aureus* and CNS Isolated in This Study

Eleven (11) antibiotics belonging to different classes were tested on *S. aureus* and coagulase negative *S. aureus*. Of 49 *Staphylococcus aurei* isolated, most were resistant against Cefazolin (34.7%), Cefoxitin (34.7%), Cloxacillin (34.7%), Augmentin (34.7%), Tetracycline (34.7%), and Erythromycin (22.4%). Some *S. aureus* were also resistant against Clindamycin (12.2%), Fusidic acid (8.2%), and Gentamicin (4.1%). However, none were resistant to Linezolid.

Of the six coagulase negative *S. aureus* isolates, majority were resistant against the tested antibiotics including Cefazolin (66.7%), Cefoxitin (66.7%), Clindamycin (66.7%), Cloxacillin (66.7%), Augmentin (66.7%), Cotrimoxazole (66.7%), Erythromycin (66.7%), tetracycline (66.7%), Fusidic acid (50.0%) and Gentamicin (50.0%). However, none were resistant to Linezolid (Table 4).

**Table 4:** Antibiogram of *S. aureus* and CNS isolated in this study.

	*Staph aureus* (*n* = 49)		CNS (*n* = 6)	
Antibiotics	*R*	*S*	*R*	*S*
Cefazolin	17 (34.7)	32 (65.3)	4 (66.7)	2 (33.3)
Cefoxitin	17 (34.7)	32 (65.3)	4 (66.7)	2 (33.3)
Clindamycin	6 (12.2)	43 (87.8)	4 (66.7)	2 (33.3)
Cloxacillin	17 (34.7)	32 (65.3)	4 (66.7)	2 (33.3)
Augmentin	17 (34.7)	32 (65.3)	4 (66.7)	2 (33.3)
Cotrimoxazole	5 (10.2)	44 (89.8)	4 (66.7)	2 (33.3)
Erythromycin	11 (22.4)	38 (77.6)	4 (66.7)	2 (33.3)
Fusidic Acid	4 (8.2)	45 (91.8)	3 (50.0)	3 (50.0)
Gentamycin	2 (4.1)	47 (95.9)	3 (50.0)	3 (50.0)
Tetracycline	17 (34.7)	32 (65.3)	4 (66.7)	2 (33.3)
Linezolid	0 (0.0)	49 (100.0)	0 (0.0)	6 (100.0)

*Note:* Data presented as frequency and percentage.

## 4 Discussion

The present study provides insight into the prevalence and antibiotic resistance patterns of wound infection‐associated bacterial pathogens, revealing a high burden of resistance to widely used antimicrobial agents. Among organisms causing wound infections isolated, most were *E. coli* (23.2%), followed by *P. aeruginosa* (19.8%), *Klebsiella spp*. (19.2%), and *S. aureus* (15.2%) is consistent with the study of Raza et al. [18] in Nepal, who found *S. aureus* (37.5%) was the predominant Gram‐positive isolate, and *E. coli* (25%) was the major Gram‐negative isolate in wound infections [18]. Similarly, Maharjan et al. reported *S. aureus* (48.3%) was the most predominant Gram‐positive bacterium isolated, and *E. coli* (16.3%), and *K. pneumoniae* were the predominant Gram‐negative bacteria isolated in wound infections (*n* = 23, 8.5%) [19]. Narula et al. [20] study also reported the most common organism isolated in wound infections was *S. aureus*, followed by *K. pneumoniae* [20]. In addition, studies by Pondei et al. [21] in Nigeria and Shimekaw et al. [22] among patients with wound infections in Ethiopia showed that most bacteria were *S. aureus,* which was the most frequently isolated Gram‐positive bacterium, accounting for 32.1% of isolates, and *P. aeruginosa* accounted for 15.4% of Gram‐negative bacilli, being the most prevalent pathogen isolated [21, 22]. This study also observed that a significant number of *Acinetobacter baumani* (6.2%), *Candida* spp. (5.0%), *Proteus* spp. (4.0%), and *Enterobacter* spp. (2.5%) also contributes to wound infection among Ghanaians. Interestingly, the distribution of isolated organisms causing wound infections was significantly different between males and females. This calls for extra monitoring of wound infection organisms for effective treatment options.

Susceptibility outcome revealed that aminoglycoside (Amikacin and Gentamicin), carbapenem (Ertapenem, Imipenem, and Meropenem) and tetracycline (Tigecycline) were the most effective antibiotic against Gram negative bacteria in wound infections whilst 2nd, 3rd and 4th generation cephalosporin (Cefuroxime, Ceftriaxone, and Cefepime, respectively), and aminopenicillin (Amoxicillin) had the highest resistant to Gram negative bacteria in wound infections. Moreover, Gram‐positive bacteria such as *S. aureus* and coagulase‐negative *S. aureus* (CNS) were most susceptible to aminoglycosides (Gentamicin), lincosamides (Clindamycin), and macrolides (Erythromycin), however, they had the highest resistance against 1st and 2nd generation cephalosporin (Cefazolin and Cefoxitin, respectively), penicillin (Augmentin and Cloxacillin), and Tetracycline. In line with these study findings, Anthony et al. [23] reported aminoglycosides were effective against both Gram‐positive and Gram‐negative bacteria, and AMR was higher among Gram‐negative isolates as compared to the Gram‐positive bacteria [23]. Narula et al. [20] study also showed most Gram‐positive isolates were resistant to penicillin and cephalosporin antibiotics and were moderately susceptible to aminoglycosides [20]. Moreover, Maharjan et al. (2020) study showed Gentamicin followed by co‐trimoxazole was the most effective among the tested antibiotics for Staphylococcus aureus, and Gentamicin and ciprofloxacin were also effective for isolated gram‐negative bacteria [19]. This study finding also confirms that of Bayram et al. [24] that multidrug‐resistance has emerged as an important concern, and Tigecycline was found to be the most active drug against *A. baumannii,* whilst Carbapenems and amikacin were found to be the most active drugs against other Gram‐negative bacteria; and Vancomycin and linezolid were active against Gram‐positive bacteria [24]. In addition, Abdu et al. [25] also showed Amikacin as the most efficacious for managing wound infections, with all the isolates being susceptible to it [25].

This study found that the prevalence of ESBL‐producing organisms isolated was 30.3%. Moreover, the majority of the ESBL‐producing organisms were *E. coli* (50.0%) and Klebsiella spp. (43.9%). In line with this study, Narula et al. [20] showed that the rising resistance to penicillin and cephalosporin group of antibiotics could be due to the high prevalence of extended‐spectrum beta‐lactamases (ESBLs) producing organisms, which is an alarming situation [20]. Maharjan et al.'s study found 20.7% of total Enterobacteriaceae were extended‐spectrum beta‐lactamase producers [19]. The observed finding calls for enhanced treatment options for ESBL‐producing organisms in order to reduce antimicrobial resistance among the Ghanaian population.

The prevalence of MRSA‐positive organisms (34.7%) found in the study is comparable to other studies' reported incidence of MRSA, ranging from 20% to 56.5% [20, 26]. Furthermore, the prevalence of methicillin‐resistant coagulase‐negative *S. aureus* was 66.7%. In line with this finding, Raza et al. [18] study in Nepal found that out of 36 *S. aureus*, 41.66% isolates were methicillin‐resistant *S. aureus* (MRSA) [18, 20]. In addition, Maharjan et al.'s study found 44.6% of total Staphylococcus aureus were Methicillin‐resistant *S. aureus* positive [19]. The antibiogram of Gram‐positive isolates, including *S. aureus* and CNS, showed the highest resistance against Cefazolin, Cefoxitin, Cloxacillin, Augmentin, Tetracycline, and Erythromycin. Other studies also support a rise in resistance against penicillin and cephalosporin [20, 27]. The rising resistance to penicillin and the cephalosporin group of antibiotics could be due to the overuse of these drugs and the high prevalence of extended‐spectrum beta‐lactamases (ESBLs) producing organisms. Other studies also support the gradual increase in the emergence of antibiotic‐resistant microorganisms [28, 29]. To decrease the emergence of such multidrug‐resistant organisms, all three important factors, such as host factors, microbial factors, and environmental factors, must be considered.

## 5 Conclusion

The most isolated organisms causing wound infections are *E. coli*, *P. aeruginosa*, *Klebsiella* spp., and *S. aureus*. Aminoglycosides (Amikacin and Gentamicin) and Linezolid are most effective against Gram‐positive and Gram‐negative bacteria causing wound infections. However, both Gram‐positive and Gram‐negative bacteria are resistant to 2nd generation cephalosporins and penicillin. The substantial prevalence of ESBL‐producing organisms and methicillin‐resistant *S. aureus* further highlights a growing antimicrobial resistance burden in wound infections. These findings point to the need for routine culture and antimicrobial susceptibility testing to guide appropriate therapy. Future studies should focus on continuous antimicrobial resistance surveillance, molecular characterization of resistance mechanisms, and the strengthening of antimicrobial stewardship programs to improve wound infection management and limit the spread of resistant pathogens in Ghana.

## 6 Recommendations

The rising resistance to cephalosporin and penicillin group of antibiotics could be due to the overuse of these drugs and the high prevalence of extended‐spectrum beta‐lactamases (ESBLs) producing organisms. This calls for effective awareness of antimicrobial resistance and the reduction of antibiotic use for non‐medical purposes among Ghanaians. Thus, to tackle the high antibiotic resistance rate, health education, interruption of illegal drug healing practices, and irrational drug prescription in health facilities should be promoted.

Moreover, in the absence of culture and drug susceptibility testing, aminoglycosides (Amikacin and Gentamicin), and carbapenems (Ertapenem, Imipenem, and Meropenem) can be used in preference to other commonly used antibiotics for the treatment of wound infections in the study area. Further study should be done by including anaerobic bacteria and fungi.

## Author Contributions

N.K., S.A.B., and E.S. were involved in conceptualization and project administration. N.K. and E.S. were involved in data curation and statistical analyses. N.K., S.A.B., L.A., O.L.A., U.P., S.A., and E.S. were involved in study design, investigation, manuscript drafting, and reviewing. All authors have approved the final version of the manuscript.

## Funding

The author received no specific funding for this work.

## Ethics Statement

Ethical approval was obtained from the ethics and protocol review committee of Narh Bita College before the study was conducted. The study was conducted following the guidelines of the Helsinki Declaration.

## Consent

A detailed explanation of the study protocol and assurance of anonymity were provided to the subjects. Participants were then given written informed consent, which was read and explained to them in a language they understood, to consent before participating in this study.

## Conflicts of Interest

The authors declare no conflicts of interest.

## Transparency Statement

The corresponding author, Ebenezer Senu, affirms that this manuscript is an honest, accurate, and transparent account of the study being reported; that no important aspects of the study have been omitted; and that any discrepancies from the study as planned (and, if relevant, registered) have been explained.

## Data Availability

All data generated or analyzed during this study are included in this article and can be requested from the corresponding author.
